# Recent Advances in Encapsulation for Food Applications

**DOI:** 10.3390/foods13040579

**Published:** 2024-02-14

**Authors:** Berta Nogueiro Estevinho, Amparo López-Rubio

**Affiliations:** 1 LEPABE—Laboratory for Process Engineering, Environment, Biotechnology and Energy, Departamento de Engenharia Química, Faculdade de Engenharia da Universidade do Porto, Rua Dr. Roberto Frias, 4200-465 Porto, Portugal; 2 ALiCE—Associate Laboratory in Chemical Engineering, Faculty of Engineering, University of Porto, Rua Dr. Roberto Frias, 4200-465 Porto, Portugal; 3 Food Safety and Preservation Department, CSIC—Consejo Superior de Investigaciones Científicas, Instituto de Agroquimica y Tecnologia de los Alimentos (IATA), 46980 Paterna, Valencia, Spain

Food-related research is closely related to health. The close relationship between food and health has also been translated to consumers, who now not only pay more attention to their lifestyles and diets but are also aware that the food they consume could play a role in preventing chronic diseases and improving their health. In this sense, food industries are interested in developing new food matrices using modern solutions and techniques [1]. Encapsulation has become an interesting tool to boost functional food design with biotechnological and bioactive applications, which is of significant interest to the food industry [2,3,4]. Encapsulation is a technology used for protecting certain substances (like bioactive molecules) and allowing their controlled release, especially in matrices in which these substances could not otherwise be incorporated [5,6,7].

The development of these new fortified foods and beverages containing a variety of bioactive compounds is being investigated to take advantage of their health benefits [8,9,10]. However, it is widely known that some of these bioactive compounds are incredibly sensitive to ambient or industrial processing conditions [11,12]. Some of these compounds are very volatile; they can also react with other components, and many of them are susceptible to heat and moisture. In order to tackle these issues, encapsulation tools have been explored with the objective of not only protecting against the environmental factors previously mentioned but also aiming to protect their bioactivity and/or improve their bioaccessibility and bioavailability when exposed to internal body conditions, i.e., after ingestion [13]. Therefore, encapsulation is an attractive technique to entrap and protect unstable active compounds (core materials) within different polymer matrices (shell materials) [14,15,16]. Encapsulation processes, as previously mentioned, can be used to protect the core compounds and to reduce their reactivity with other substances. Encapsulation also decreases the transfer rate of the active compound from the core to the outside, controlling, in this way, the release of the inner substance. Another advantage is that it can make handling easier and can even mask potential strong tastes and odors, thus improving the sensorial properties of the food products [17].

This Special Issue compiles some recent and innovative research results regarding the encapsulation of active and natural compounds, seeking to provide a fundamental understanding of the process and the current strategies employed to improve the encapsulation of specific compounds such as flavors, vitamins, stabilizers, probiotics, essential oils, natural antioxidants, bioactive proteins and enzymes, among others.

Specifically, this Special Issue comprises five research articles that explore novel encapsulation techniques or encapsulation matrices, physicochemical characteristics of the encapsulation systems and their food applications, including sensorial characteristics, the nutritional value of food products containing encapsulated compounds and/or their simulated digestion behavior.

Two of the papers presented in this Special Issue are related to the encapsulation of a catechin—epigallocatechin gallate (EGCG)—considering that it is one of the most abundant polyphenols in green tea and has been broadly studied due to its potential benefits to human health, including antitumor, antiradiation and anti-oxidant effects [18]. However, direct incorporation within food matrices has been limited because of its low oral bioavailability and poor stability. The manuscript by Jin et al. describes the use of zein–gum arabic complex nanoparticles to protect this tea polyphenol, and the authors studied the stability of the compound upon in vitro simulated digestion [19]. Zein and gum arabic (GA) were used as wall materials to prepare zein–GA complex nanoparticles for encapsulating and protecting the EGCG. The nanoparticles obtained were characterized in terms of particle size, polydispersity index (PDI), zeta potential, encapsulation efficiency and loading capacity (LC). A broad range of techniques was used for characterization, showing average particle sizes of the zein–GA–EGCG complex nanoparticles of around 200 nm, with good efficiency, reaching more than 75% when the mass ratio of zein to GA was 1:1. The release rate of EGCG was also evaluated during gastric digestion, showing that the encapsulation system adequately preserved the polyphenol molecules, with a large amount (more than 70%) retained during the gastric phase, thus suggesting they were protected during digestion. The authors concluded that zein–GA complexes have great potential as encapsulation matrices to protect EGCG during simulated gastrointestinal digestion [19].

In another article, Ralaivao et al. [18] described the encapsulation of EGCG using various food-grade matrices including gum arabic, modified chitosan and sodium alginate. The authors adopted a spray drying method. In this case, instead of nanoparticles, the average capsule size was in the micron range (between ~4 and 40 µm), and the matrices used also affected capsule morphologies and surface roughness. The encapsulation efficiency was also very good, ranging between 78.5 and 100.0%. The EGCG release was evaluated in deionized water, and a quick release was observed. The release profiles were adjusted using three kinetic models: Korsmeyer–Peppas, Weibull and Baker–Lonsdale. The Weibull model was the superior model for describing EGCG release. The authors also considered the ideal daily amount of EGCG uptake required to avoid secondary effects, such as the risk of hepatotoxicity, reporting 800 mg EGCG/day as the “safety” dose; they also determined the amount of powder (number of microparticles) that could be ingested as a food supplement or additive per day. The authors concluded that EGCG microencapsulation using the spray drying technique was successful [18].

Another two papers of this Special Issue also made use of the spray drying technique to produce microparticles containing different bioactive compounds. Spray drying is a relatively low-cost technology, widely used at the industrial scale due to its high production rates; these factors have encouraged research investigating the transfer of this technology for other purposes. One of the manuscripts by Zhao et al. explored the microencapsulation of garlic essential oil [20]. This essential oil has antibacterial and antioxidant properties, and it is also used as a flavoring agent. However, the active components in garlic essential oil are easily degraded, thus limiting its direct application within food products. Zhao et al. blended this garlic essential oil with various vegetable oils (corn oil, soybean oil and olive oil) to improve its stability [20]. It was found that adding an appropriate amount of vegetable oil promoted the stability of the garlic essential oil emulsion. Evaluation of the encapsulation efficiency, controlled release and antimicrobial activity of the microcapsules showed that the garlic essential oil was successfully entrapped and slowly released, as well as exhibiting active antibacterial activities on both *E. coli* and *S. aureus*. The authors concluded that the vegetable oils, especially 20% corn oil, improved the stability of garlic essential oil emulsions and the encapsulation efficiency of garlic essential oil microcapsules.

The other manuscript in which spray drying was used for microencapsulation aimed to incorporate the enzyme Flavourzyme^®^ within chitosan capsules [21]. Chitosan has been widely explored for enzyme immobilization, as this polysaccharide is cheap, non-toxic and has high protein affinity [22]. Flavourzyme^®^ is a fungal protease–peptidase complex derived from *Aspergillus oryzae* and used for the acceleration of cheese ripening, particularly for the debittering and flavor development of cheese [21]. Crosslinking of the capsules with TPP improved encapsulation efficiency and protein stability, and the effect of enzyme concentration and operational conditions (including the drying temperature) were also evaluated. In this regard, an activity yield of 88.0% and an encapsulation efficiency of 78.6% were obtained with a concentration of 0.1% (*v*/*v*) and an inlet temperature of 130 °C. The microcapsules were also characterized in terms of their size and morphology using scanning electron microscopy and laser diffractometry. The authors concluded that Flavourzyme^®^ was successfully encapsulated within the crosslinked chitosan matrix with high protease activity retention, thus making it an interesting option for the application of this commercial protease in food processing, e.g., the acceleration of cheese ripening [21].

The last paper published in this Special Issue focused on a different encapsulation technique to develop micro- or nanostructures for the delivery of active compounds—electrohydrodynamic processing (EHD) [23]. The EHD technique includes two variations: electrospinning and electrospraying. In these processing technologies, a polymer solution can either be spun or sprayed through the application of an external electric field, giving rise to fibers or particles, respectively. In this manuscript, Tomadoni et al. prepared electrosprayed agar nanocapsules as edible carriers of a model bioactive compound (chlorophyllin sodium copper salt) [23]. These nanocapsules were developed using an acetic acid solution as a solvent, and the role of the solution’s properties in the formation of agar particles was evaluated in terms of capsule size, morphology and encapsulation efficiency. The release profile of the chlorophyllin sodium copper salt was studied using two food simulants with different hydrophilicity (10% *v*/*v* and 50% *v*/*v* ethanol). The authors concluded that the release of the bioactive compound was negligible in the hydrophilic food simulant, while an initial intense release followed by a slower, sustained release was observed when the capsules were immersed in 50% ethanol solution [23].

The papers presented in this Special Issue represent a small part of the research that is ongoing in the field of encapsulation of bioactive compounds for food applications all over the world. With outstanding results and much more potential to explore in the coming years, microencapsulation techniques are promising candidates in the creation and reformulation of new food products. The editors anticipate that this Special Issue will stimulate readers in the field and contribute new ideas or methodologies for their future work.
